# Overexpression of a natural chloroplast-encoded antisense RNA in tobacco destabilizes 5S rRNA and retards plant growth

**DOI:** 10.1186/1471-2229-10-213

**Published:** 2010-09-29

**Authors:** Amber M Hotto, Zoe E Huston, David B Stern

**Affiliations:** 1 Boyce Thompson Institute for Plant Research, Cornell University, Tower Rd., Ithaca, NY 14853, USA; 2 Riverdale High School, 9727 SW Terwilliger Blvd., Portland, OR 97219, USA

## Abstract

**Background:**

The roles of non-coding RNAs in regulating gene expression have been extensively studied in both prokaryotes and eukaryotes, however few reports exist as to their roles in organellar gene regulation. Evidence for accumulation of natural antisense RNAs (asRNAs) in chloroplasts comes from the expressed sequence tag database and cDNA libraries, while functional data have been largely obtained from artificial asRNAs. In this study, we used *Nicotiana tabacum *to investigate the effect on sense strand transcripts of overexpressing a natural chloroplast asRNA, AS5, which is complementary to the region which encodes the 5S rRNA and tRNA^Arg^.

**Results:**

AS5-overexpressing (AS5^ox^) plants obtained by chloroplast transformation exhibited slower growth and slightly pale green leaves. Analysis of AS5 transcripts revealed four distinct species in wild-type (WT) and AS5^ox ^plants, and additional AS5^ox^-specific products. Of the corresponding sense strand transcripts, tRNA^Arg ^overaccumulated several-fold in transgenic plants whereas 5S rRNA was unaffected. However, run-on transcription showed that the 5S-*trnR *region was transcribed four-fold more in the AS5^ox ^plants compared to WT, indicating that overexpression of AS5 was associated with decreased stability of 5S rRNA. In addition, polysome analysis of the transformants showed less 5S rRNA and *rbcL *mRNA associated with ribosomes.

**Conclusions:**

Our results suggest that AS5 can modulate 5S rRNA levels, giving it the potential to affect Chloroplast translation and plant growth. More globally, overexpression of asRNAs via chloroplast transformation may be a useful strategy for defining their functions.

## Background

Chloroplasts originated around 1.5 billion years ago via an endosymbiotic event where a primitive eukaryote engulfed an ancestor of modern-day cyanobacteria. Subsequently, massive gene transfer to the nucleus occurred, resulting in a highly reduced plastid genome of 120-160 kb that possesses ~120 genes. These remaining genes are mostly organized into clusters, and their expression requires a combination of prokaryotic and eukaryotic-like post-transcriptional events including maturation of polycistronic transcripts, splicing, RNA editing, and 5' and 3' end trimming [reviewed in [1]]. These processes are catalyzed by nucleus-encoded proteins, many of which were originally encoded by the chloroplast ancestor [2].

Post-transcriptional regulation in both prokaryotes and eukaryotes is also exerted by non-coding RNAs (ncRNAs), which include antisense RNAs (asRNAs) and transcribed intergenic sequences. The asRNAs can act in *cis*, on the cognizant sense strand transcript, or in *trans*, targeting one or more distantly-located genes through specific base pairing. Antisense RNAs regulate many steps in gene expression including translation initiation, mRNA stability, alternative splicing, RNA editing, and transcription termination [3,4]. Clear evidence exists that asRNAs are prevalent in cyanobacteria, the prokaryote most closely related to chloroplasts. In *Prochlorococcus *MED4 and *Synechocystis *sp. PCC 6803, asRNAs were found using microarrays, and some were verified by RNA blot and shown to be differentially regulated under altered stress and nutrient conditions [5,6]. Functions have been ascribed to two cyanobacterial asRNAs, α-*furA *and IsrR, which act in *cis *to occlude a ribosomal binding site and regulate sense strand transcript accumulation, respectively [7,8]. Thus, the chloroplast progenitor likely used asRNAs for gene regulation, suggesting that at least part of this capacity might have been retained in the present-day organelle.

The occurrence of organellar ncRNAs has been established experimentally from sequencing of cDNA libraries [9,10]. In one study, ncRNAs were abundantly detected in plant mitochondria deficient for polynucleotide phosphorylase (PNPase), a 3' to 5' exoribonuclease involved in RNA maturation and decay, some of which were also found in wild-type (WT) plants [1,9]. In chloroplasts, sequencing of small RNAs identified a number of ncRNAs [10,11]. Although their functions were not directly tested, at least some of them appear to be footprints of sequence-specific RNA-binding proteins involved in mRNA 3' end formation [12]. More recently, an RNA antisense to the chloroplast *ndhB *gene in *Arabidopsis*, *Nicotiana tabacum *and poplar was identified, which was hypothesized to regulate RNA maturation or stability [13]. Direct evidence for chloroplast asRNA function comes primarily from two studies, one in which an ectopically expressed asRNA resulted in reduced C-to-U editing in tobacco [14], and a second where a chloroplast genome rearrangement in *Chlamydomonas *led to expression of an asRNA that stabilized the corresponding sense strand transcript [15].

The present study focuses on AS5, a chloroplast-encoded asRNA that is represented by several ESTs (see below), and was found to be highly abundant in the *Arabidopsis rnr1-3 *mutant (Sharwood, Hotto, Bollenbach and Stern, unpublished results). This mutant lacks ribonuclease R (RNR1), a 3' to 5' exoribonuclease, which is dually targeted to mitochondria and chloroplasts [16,17], and whose prokaryotic orthologue is involved in rRNA maturation and the degradation of structured RNAs [18,19]. The *rnr1-3 *mutant accumulates precursors for several transcripts encoded in the chloroplast ribosomal operon, but is particularly deficient in the accumulation of both precursor and mature forms of 5S rRNA, which is encoded by the *rrn5 *gene that lies just upstream of *trnR*, which encodes tRNA^Arg^. Because AS5 is antisense to the 5S-*trnR *region, and because *rnr1-3 *overaccumulates AS5 and underaccumulates the complementary 5S rRNA, we postulated that AS5 might regulate 5S rRNA processing or stability. Here we have overexpressed AS5 in tobacco (*Nicotiana tabacum*) chloroplasts to determine its *in vivo *function in the absence of any pleiotropic effects caused by RNR1 deficiency. The results show that overepxressing AS5 leads to a slow-growth phenotype and decreased 5S rRNA stability, suggesting a possible function for AS5 in WT chloroplasts.

## Results

### Creation of tobacco chloroplast transformants

**Figure 1:**
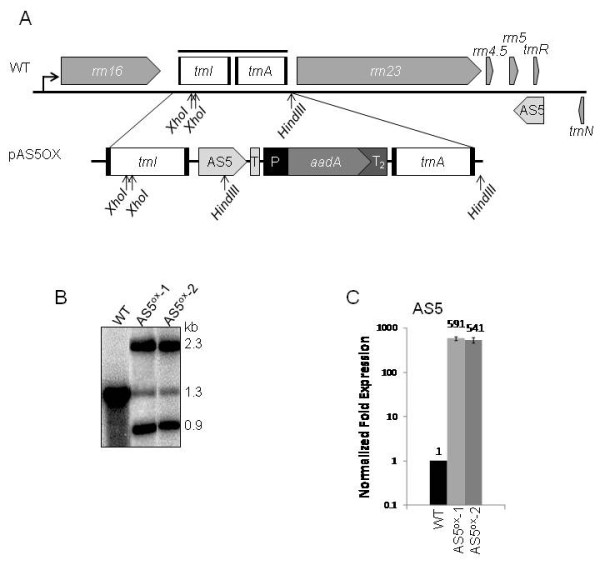
**Creation and validation of two AS5^ox ^lines**. (A) Diagram of the native tobacco chloroplast *rrn *operon (WT) with the transgene insertion site. The plastid transformation vector (pAS5OX) is shown below (T-*psbA *terminator; P-*psbA *promoter; T_2_-*rps16 *terminator). Positions of the *Xho*I and *Hind*III restriction sites, and the DNA probe (solid line) used in (B) are indicated. (B) DNA gel blot of WT and two AS5^ox ^lines digested with *Xho*I and *Hind*III. A PCR fragment spanning the *trnI *and *trnA *region was used as a probe. Relevant sizes of hybridizing bands are shown at the right. (C) Quantitative RT-PCR analysis of AS5 transcripts using primers AS5 qPCR 5' and 3'. Expression levels are an average of three biological and at least two technical replicates of each sample, with error bars representing the standard deviation. The WT expression level was set to 1, and samples were normalized to 18S rRNA and GAPDH mRNA.

Transplastomic tobacco plants were obtained using the vector pAS5OX, which resulted in the overexpression of AS5 (Figure 1A). This vector targets the *trnI-trnA *region in the chloroplast ribosomal (*rrn*) operon located in the inverted repeat region through homologous recombination, and relies on readthrough from the upstream P_rrn _promoter for AS5 transcription. This insertion site is well-characterized and has not been reported to affect plant growth and development [reviewed in [20]]. The selectable marker *aadA *possesses its own promoter and is transcribed in tandem with the *rrn *operon. As shown below, this leads to enhanced transcription of genes downstream of *aadA*. The AS5 sequence expressed from the transformation vector is the antisense strand extending from just downstream of the *trnR *gene, to the 3' end of *rrn5*. Endogenous AS5 is also expressed from this operon, albeit on the opposite strand.

Chloroplast transformants were identified by growth on selective medium and PCR (Experimental Procedures). After 3-4 rounds of regeneration, the transgenic lines were further validated by DNA gel blot after restriction digest with *Xho*I and *Hind*III (Figures 1A &1B). The *Xho*I site is within the *trnI *intron, while *Hind*III cleaves in the middle of the AS5 transgene and just downstream of *trnA*. Two independent AS5 overexpression (AS5^ox^) lines were confirmed (AS5^ox^-1 and AS5^ox^-2), with expected bands of 2.3 and 0.9 kb (Figure 1B). Surprisingly, both lines retained a weak band at 1.3 kb, corresponding to a WT-like size. This is likely due to a nuclear-encoded fragment, rather than a plastome sequence, and has been seen in other chloroplast transformants targeting the *rrn *operon and other regions [21,22]. Furthermore, an identical pattern was observed after the plants had been self-crossed, which normally resolves any residual heteroplasmy. To verify that increased AS5 expression was occurring in the AS5^ox ^lines, we used quantitative RT-PCR (qRT-PCR; Figure 1C). After normalization to two nuclear control mRNAs, we found that the AS5^ox ^lines accumulated >500-fold more AS5, confirming the expected transgene expression.

### Plant growth is slowed in the AS5^ox ^lines

**Figure 2:**
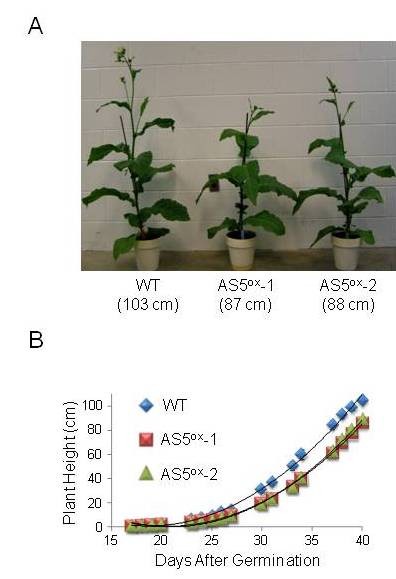
**Growth analysis of WT and transplastomic plants**. (A) Representative WT and transplastomic plants 40 days after germination on soil. Plant heights are indicated in parentheses. (B) Growth curve of WT and transplastomic plants from 16-40 days after germination.

Growth was compared between AS5^ox^-1, AS5^ox^-2 and WT tobacco grown in the greenhouse. The AS5^ox ^lines exhibited slower growth beginning 19 days after germination, which resulted in delayed flowering time (Table 1; Figure 2). Forty days after germination, we observed that internode 3 was not significantly reduced in length, while internode 9 was shorter. In addition, the stem circumference at both internodes was significantly reduced (~7% decreased at internode 3, and ~16% decreased at internode 9). The differences in internode length and width resulted in shorter plants with lighter shoots after 40 days. Shoot fresh weight at maturity was reduced by about 10% compared to the WT, which can likely be ascribed to reduced stem diameter, as the plant height at maturity did not differ (data not shown). Leaf 3 and leaf 9 dimensions and weight on a per area basis were measured 40 days after germination. Leaf 3, near the base of the plant, was not significantly altered in length, width or fresh weight, while the dry mass was slightly increased in the AS5^ox ^plants. However, leaf 9 was significantly smaller in both length and width, while unit area weight was unaffected. Lastly, total chlorophyll was reduced by ~10% in the AS5^ox ^lines at maturity, corresponding to a slightly pale-green leaf phenotype. Taken together, these data indicate that the overall vigor of the AS5^ox ^lines is diminished, especially with respect to stem elongation.

**Table 1:** Phenotypic characteristics of AS5^ox ^lines.

		WT	AS5^ox^-1	AS5^ox^-2
*Days to Flowering from Planting		50.60 ± 1.43	53.80 ± 0.79	53.80 ± 0.79
*Total Chlorophyll (g/m^2^) at Maturity		0.75 ± 0.05	0.69 ± 0.04	0.66 ± 0.04

*Plant Height (cm)		99.97 ± 1.65	81.46 ± 2.36	86.66 ± 1.90
*Shoot Fresh Weight (g)		101.69 ± 3.02	82.39 ± 3.29	83.21 ± 3.04

Internode 3	Length (cm)	5.71 ± 0.19	5.37 ± 0.24	5.12 ± 0.29
	*Circumference (cm)	4.44 ± 0.05	4.06 ± 0.06	4.16 ± 0.09

Internode 9	*Length (cm)	15.98 ± 0.49	12.61 ± 0.61	13.27 ± 0.78
	*Circumference (cm)	3.98 ± 0.10	3.16 ± 0.12	3.52 ± 0.11

Leaf 3	Length (cm)	30.77 ± 0.77	32.17 ± 1.22	30.11 ± 0.95
	Width (cm)	19.85 ± 0.47	21.04 ± 0.95	19.82 ± 0.69
	Fresh Weight (mg/cm^2^)	20.53 ± 0.79	17.16 ± 0.43	20.23 ± 0.73
	*Dry Weight (mg/cm^2^)	1.68 ± 0.07	1.80 ± 0.04	1.95 ± 0.06

Leaf 9	*Length (cm)	33.82 ± 1.25	27.10 ± 1.92	25.02 ± 1.42
	*Width (cm)	16.34 ± 0.66	11.93 ± 1.27	11.01 ± 0.90
	Fresh Weight (mg/cm^2^)	14.34 ± 0.54	11.97 ± 0.17	13.99 ± 0.37
	Dry Weight (mg/cm^2^)	2.02 ± 0.07	1.86 ± 0.09	2.13 ± 0.08

Results represent an average ± the standard error of measurements taken 40 days after germination (WT - n = 18; AS5^ox^-1 and AS5^ox^-2 - n = 17) or at maturity (n = 10). Internode and leaf numbers were counted starting from the base of the plant.

*Traits with a significant difference (p < 0.05) between both AS5^ox ^lines and WT.

### AS5 is present as multiple transcripts

**Figure 3:**
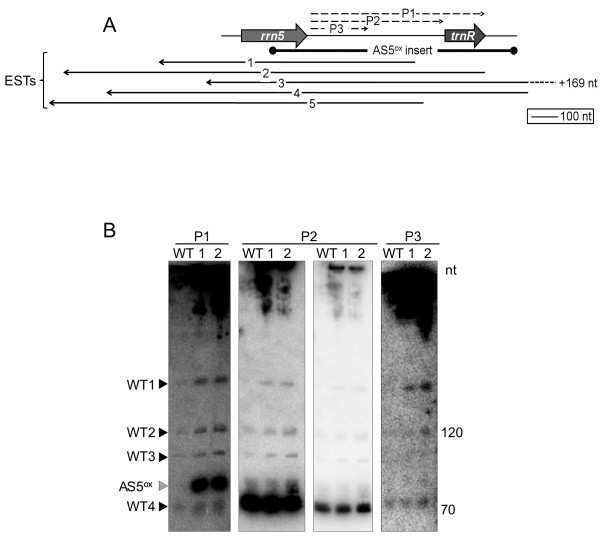
**AS5 transcript analysis**. (A) Extent of the AS5 transgene (black bar) in relation to *rrn5*, *trnR*, and representative tobacco and tomato AS5 EST's (solid black arrows). Locations of the strand-specific RNA probes (P1-P3) used in (B) are shown above (dashed black arrows). ESTs are: 1 - tobacco AM826917, 479 nt; 2 - tomato AJ85186, 785 nt; 3 - tomato BI422136, 774 nt; 4 - tomato EST587142, 787 nt; 5 - tomato AJ832014, 696 nt. (B) 10% polyacrylamide gel blot analysis of AS5 in WT and AS5^ox^-1 (1) and AS5^ox^-2 (2). The probes used are shown at the top of the blot, and four WT bands (WT1-4) and an AS5^ox^-specific band (AS5^ox^) are indicated with arrows at the left. Two exposures are shown for the P2 probe to confirm bands WT1-3 and illustrate the abundance of WT4. Size standards are shown at the right.

EST data from tobacco and its solanaceous relative tomato suggested that AS5 is present in multiple overlapping forms (Figure 3A). These ESTs, however, represent partial sequences from size-selected cDNAs, and are derived from polyadenylated transcripts, which are inherently unstable in chloroplasts [23]. To assess which discrete species might accumulate in WT or transformed plants, a series of overlapping strand-specific probes spanning the 5S-*trnR *region were used on polyacrylamide gel blots of total leaf RNA (Figure 3B).

The longest probe, P1, contained the 5S-*trnR *intergenic region and the *trnR *coding region. The resulting banding pattern revealed four AS5 transcripts of 70-400 nt in the WT (WT1-4), and an additional 80 nt species in the AS5^ox ^lines. The multiple species may result from endo- and/or exonucleolytic maturation of an AS5 primary transcript, and/or from specific cleavages of AS5 bound to a sense RNA target. Each of the four AS5 bands common to all samples overaccumulated in the AS5^ox ^lines, suggesting that at least a portion of the transgene transcript was functioning in a manner similar to the endogenous one. The AS5^ox ^lines also accumulated larger transcripts not resolved in the polyacrylamide gel (see below).

Additional probes were used to further characterize the AS5 species. Probe P2 contained only the 5S-*trnR *intergenic region and identified all four WT bands, indicating that the WT1-4 bands must contain part of the intergenic region. However, the WT4 band was most strongly detected. This increased hybridization to the WT4 band, as compared to results with P1, may reflect secondary structure differences between probes P1 and P2. The AS5^ox^-specific band was not detected with P2, demonstrating that this band contains sequences antisense to the *trnR *coding region, and possibly additional sequence derived from the transgene.

Probe P3 spans the proximal part of the 5S-*trnR *intergenic region. This probe hybridized more weakly than P1 and P2, which may be due to less sequence overlap with the targets, and/or its low (27%) G+C content compared to the full intergenic region (41%). The strongest hybridization was to WT1 and WT4, although WT2 was faintly visible. This suggests that WT1 and WT4 are fully derived from the region covered by P3. WT2 appears to have some overlap with P3, but is likely to be mostly derived from more distal portions of the intergenic sequence, whereas WT3 appears to be derived entirely from the distal part of the intergenic region. It is possible that WT1 serves as a precursor for each of the smaller transcripts, but this cannot be directly inferred. In summary, the AS5^ox ^lines overproduce the native AS5 species and at least one novel small AS5 molecule.

### AS5^ox ^lines accumulate processing intermediates of *rrn *transcripts

**Figure 4:**
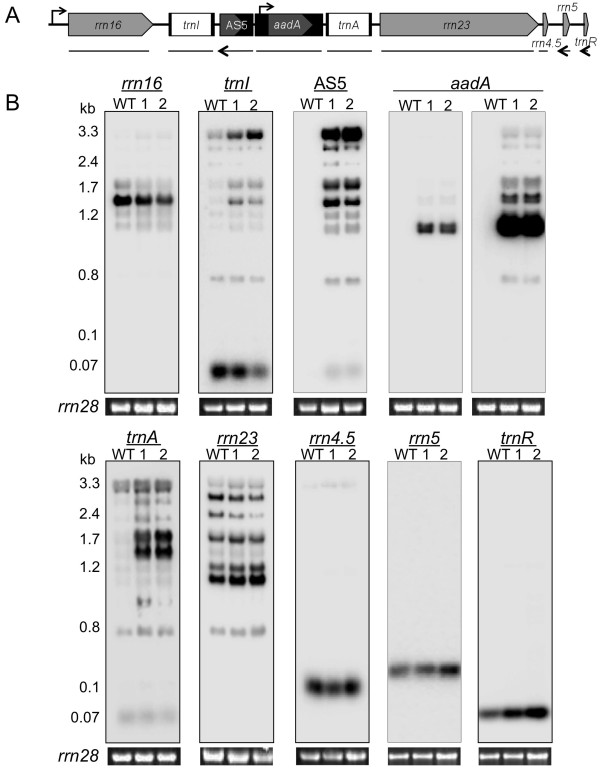
**Analysis of *rrn *operon transcripts in transplastomic and WT plants**. (A) Structure of the *rrn *operon in AS5^ox ^lines, with the endogenous and inserted promoters (bent arrows). Locations of probes used in (B) are shown beneath, with solid lines representing dsDNA probes and arrows representing RNA probes. (B) 1.2% agarose gel blot analysis, with 1 μg of total RNA from WT, AS5^ox^-1 (1) and AS5^ox^-2 (2). The probes used are indicated at the top of the blots, and estimated band sizes at the left. Two exposures are shown for the *aadA *gene, to emphasize the relative abundance of the monocistronic band. The ethidium bromide-stained 28S rRNA is shown underneath the blots to reflect loading.

Because the transgene was inserted between *trnI *and *trnA*, *rrn *operon transcripts were analyzed by RNA gel blot. The operon encodes four rRNAs and three tRNAs that are transcribed as an ~7.4 kb polycistronic precursor from a bacterial-like promoter upstream of *rrn16 *[Figure 4A; [24]]. A series of endo- and exonucleolytic steps, along with splicing of the *trnI *and *trnA *introns, yield the mature RNAs. The probes shown along the bottom of Figure 4A were used to detect any perturbations due to AS5 transgene insertion or regulation.

When blots were probed for *rrn16*, mature 16S rRNA and a precursor were detected (Figure 4B). This precursor is created by endonucleolytic cleavage in the *rrn16*-*trnI *intergenic region, leaving a 3' extension that is processed exonucleolytically [16,25]. Abundance of the mature and precursor *rrn16 *transcripts was slightly reduced in the AS5^ox ^lines.

The *trnI *probe detected mature tRNA^Ile ^(72 nt), a large polycistronic intermediate (~3.3 kb), and the ~0.8 kb unspliced *trnI *monocistron. In the AS5^ox ^lines, two additional polycistronic precursors accumulated, which appear to contain transgene sequences because they are not present in the WT and comigrate with AS5^ox^-specific AS5 bands. Unspliced *trnI *was invariant, and accumulation of mature tRNA^Ile ^decreased by ~50% in the AS5^ox ^lines. Decreased efficiency of endonucleolytic cleavage at the 3' end of *trnI*, near the transgene insertion site, may be contributing to the reduced formation of mature tRNA^Ile ^and increased accumulation of polycistronic intermediates.

The AS5 probe identified several transcripts. The largest polycistronic transcript appears to comigrate with the WT *trnI *polycistron, in addition to the two AS5^ox^-specific *trnI *bands mentioned above. Other less-abundant bands were apparent, the smallest of which migrated near tRNA^Ile ^and represents the abundant AS5^ox^-specific band detected with probe P1 in Figure 3B. We next probed for the *aadA *selectable marker transcript. The *aadA *cassette generates a monocistronic mRNA of 1.2 kb, which was by far the most abundant species. However, increased exposure revealed both longer bands and one shorter band. The former appear to be cotranscripts with *trnI *and the AS5 transgene, whereas the shorter band appears to be a cotranscript with only AS5. Thus, AS5^ox ^plants accumulate a variety of AS5-containing transcripts, some of which are the same size as endogenous species and others that are transgene-specific products. The implications of these findings for possible AS5 biological activity are considered in the Discussion.

The lower part of Figure 4B shows probes for the *rrn *operon downstream of *aadA*. Like *trnI*, the *trnA *probe revealed accumulation of additional precursors in the AS5^ox ^lines, however no effect on tRNA^Ala ^accumulation was observed. The two precursor transcripts also found in WT were a doublet at 3.2 kb and a 0.8 kb species, the latter corresponding to unspliced *trnA*. Each of these RNAs overaccumulated compared to the WT. At least five additional bands were detected in the AS5^ox ^lines, which appear to be cotranscripts with *aadA*.

Analysis of *rrn23 *transcripts revealed seven major species. This complex pattern is due to accumulation of *rrn23*-*rrn4.5 *processing intermediates and hidden breaks within the *rrn23 *transcript [26]. There were no size differences between the WT and AS5^ox ^lines and only minor quantitative differences, suggesting that there was no accumulation of *aadA *cotranscripts. Similarly, accumulation of mature 4.5S rRNA was not affected.

Finally, strand-specific probes were used to assess processing and accumulation of 5S rRNA and tRNA^Arg^. Maturation of these species involves the formation of a transient 5S-*trnR *intermediate that is cleaved by RNase P to form the 5' end of tRNA^Arg^, followed by a second endonuclease cleavage in the 5S-*trnR *intergenic region near the mature 5S 3' end [27]. The pre-5S rRNA is further matured by the 3' to 5' exonucleolytic activity of RNR1 and possibly PNPase [16]. When probed for 5S rRNA, accumulation was similar between the WT and AS5^ox ^lines. In contrast, tRNA^Arg ^overaccumulated in the AS5^ox ^lines. As discussed below, this results from increased transcription from the *aadA *promoter. No precursors were observed for either *rrn5 *or *trnR*, including when analyzed following high-resolution polyacrylamide gel electrophoresis (data not shown), indicating that maturation of their 5' and 3' ends was largely unaffected.

**Figure 5:**
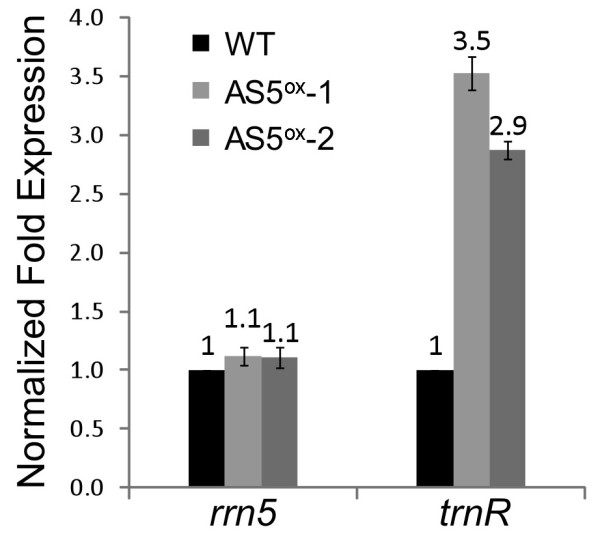
**Quantitative RT-PCR analysis of *rrn5 *and *trnR *transcripts in WT and AS5^ox ^plants**. Expression levels are an average of three biological and at least two technical replicates of each sample, with error bars representing the standard deviation. The WT expression level was set to 1, and samples were normalized to 18S rRNA and GAPDH mRNA.

Because the *rrn5 *and *trnR *transcripts were the most likely targets of regulation by the overexpressed asRNA, qRT-PCR was used to quantify their accumulation relative to two nuclear transcripts (Figure 5). As suggested by the gel blots (Figure 4B), there was only a small increase in *rrn5 *accumulation in the AS5^ox ^lines, while the *trnR *transcript increased three-fold. Overall, the results in Figures 4, 5 show that some *rrn *operon transcripts accumulated differently in the AS5^ox ^lines. This may be due to differences in transcription rates, RNA processing, and/or RNA stability.

### Increased transcription of 5S-*trnR *in the AS5^ox ^plants

**Figure 6:**
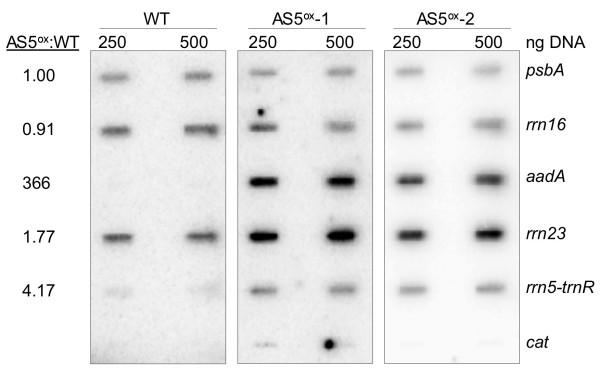
**Run-on transcription in WT and AS5^ox ^chloroplasts**. 250 or 500 ng of PCR product from chloroplast-encoded genes (right) were fixed to nylon membranes, with the chloroamphenicol acetyltransferase gene (*cat*) included as a negative control. Run-on transcription was conducted as described in Experimental Procedures. The ratio of AS5^ox ^to WT transcription (left) was calculated after background subtraction of the *cat *gene and normalization to *psbA*. The average of three experiments is shown.

We had hypothesized that AS5 regulates *rrn5 *processing and/or stability, and we observed that in the AS5^ox ^lines 5S rRNA accumulation was unaffected whereas tRNA^Arg ^was three-fold more abundant. One explanation to account for increased tRNA^Arg ^accumulation was enhanced transcription due to the upstream *aadA *promoter. This would *a priori *affect *rrn23*, *rrn4.5*, *rrn5 *and *trnR*, but not genes upstream of *aadA*, such as *rrn16*. To determine relative transcription rates in the WT and AS5^ox ^lines, a run-on assay was performed. Purified chloroplasts were labeled with α-^32^P-UTP for 5 min, after which labeled RNAs were used to probe a slot blot containing 250 and 500 ng of PCR products from rRNAs, the Photosystem II D1 protein-coding gene (*psbA*), and the chloroamphenicol acetyltransferase gene (*cat*) as a negative control (Figure 6). Because the signals did not increase in the 500 ng slots, we concluded that the target DNA was in excess over the probe. We also noted a weak signal from the *cat *gene, which was considered background noise and was subtracted from subsequent calculations. Signal intensities were normalized to *psbA*, whose transcription should be unaffected in AS5^ox ^lines.

The analysis showed that *rrn16 *transcription varied less than 10%, with the slight decrease mirroring the RNA gel blot result (Figure 4B). As *rrn16 *is proximal to the transgene promoter, a different transcription rate was not expected. When *rrn23 *was examined, the transcription rate was almost doubled in the AS5^ox ^lines, and 5S-*trnR *transcription increased four-fold. The disparity between *rrn23 *and 5S-*trnR *results may be attributed to the low signal to noise ratio for the latter in the WT, making the measurement less accurate. In any event, these results show that while transcription of *rrn5 *and *trnR *are increased in the AS5^ox ^lines, only tRNA^Arg ^increases in abundance, indicating that 5S rRNA is less stable in the AS5^ox ^lines. The unstable species could either be the mature form or a precursor with a 3' extension following *trnR *excision. In summary, increased transcription of 5S rRNA masks the instability imparted by overexpression of AS5.

### 5S rRNA polysome association decreases in AS5^ox ^lines

**Figure 7:**
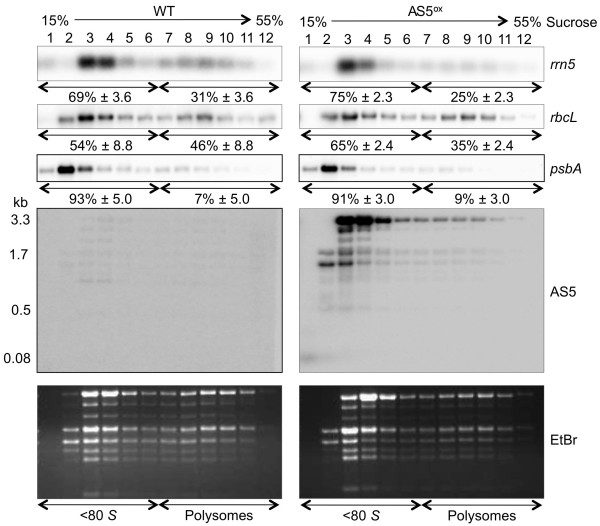
**Polysome analysis of WT and AS5^ox ^samples as described in Methods**. Radiolabeled probes used for *rrn5*, *rbcL *and *psbA *detection were dsDNA, while an RNA probe was used for AS5 detection. Estimated band sizes for AS5 are shown to the left. The ethidium bromide-stained gel represents equal proportional loading of the 12 gradient fractions. EDTA and puromycin controls (not shown) were used to determine the free ribosomal fractions (< 80*S*, lanes 1-6) and polysomal fractions (lanes 7-12). The percent of total RNA from polysomal and non-polysomal fractions are shown below the respective blots, as determined from averaging four (WT) or eight (AS5^ox^-1 and -2) experiments ± the standard error.

The AS5^ox ^lines have a slow-growth phenotype and decreased 5S rRNA stability. If AS5 were destabilizing mature or precursor 5S rRNA, it might also interfere with its incorporation into mature ribosomes. Polysome analysis was therefore conducted to assess ribosomal association of 5S rRNA and two representative mRNAs. As shown in Figure 7, polysome fractions were analyzed for *rrn5*, *rbcL*, *psbA *and AS5. The *rrn5 *probe revealed that a large portion of mature 5S rRNA is not associated with polysomes in either WT or AS5^ox ^lines. This was expected, since 5S rRNA exists in a dynamic relationship with the 50S ribosomal subunit, an association thought to influence ribosome performance [28]. In the AS5^ox ^lines, however, there was a small but distinctive and reproducible shift of 5S rRNA towards less dense fractions (lanes 1-6), with about one quarter being present in the polysome fractions (lanes 7-12). This may be an indirect influence of AS5 overaccumulation (see Discussion).

Polysomal loading of the *rbcL *and *psbA *mRNAs were investigated to identify any effect on chloroplast translational activity. As was the case for 5S rRNA, the *rbcL *mRNA shifted modestly towards the nonpolysomal fractions in the AS5^ox ^lines, suggesting that its translation rate had decreased, in keeping with their slower growth phenotype. The polysomal loading of *psbA *mRNA was not appreciably altered between WT and AS5^ox ^lines. However, this transcript is mostly nonpolysomal even in WT plants, except under conditions where PSII repair is induced [e.g. [29]].

Finally, the AS5 transcript itself was examined. Considering the data shown in Figure 4B for total RNA, we expected to observe approximately six bands between 80 nt and 3.3 kb, with the smaller species being only visible when polyacrylamide gels were used (Figure 3). Figure 7 shows that the vast majority of AS5 RNAs in the AS5^ox ^lines migrated in the nonpolysomal fractions. This result was largely expected for an ncRNA, however we noted that a portion of the longer AS5 transcripts were found in heavier fractions. Intriguingly, their accumulation pattern roughly parallels that of 5S rRNA. This raises the possibility that AS5 may bind to *rrn5 *that is incorporated into polysomes, although the hybridization results do not demonstrate that the two RNAs are actually associated. The 80 nt AS5 band found only in the AS5^ox ^lines was in fraction 1, at the top of the gradient. The other small AS5 bands are not visible in this gel system. Faint higher molecular weight bands visible on the WT blot were judged to be rRNA artifacts based on their migrations.

## Discussion

NcRNAs have been detected in both mitochondria and chloroplasts, yet there is little knowledge of their functional significance. In this study, the chloroplast asRNA AS5 was investigated for possible *cis*-acting regulation of *rrn5 *and *trnR*. We used the approach of chloroplast transformation to overexpress AS5, a strategy not previously used for a natural chloroplast asRNA. However, overexpression of nuclear-encoded miRNA is routinely used to confirm miRNA targets [reviewed in [30,31]]. While transgenes can create pleiotropic effects, we were able to correlate increased AS5 expression with decreased 5S rRNA stability and slightly reduced polysome association. While the underlying mechanism remains to be elucidated, we speculate that AS5 may restrict 5S rRNA ribosomal incorporation and/or pair with its precursor, targeting the duplex for degradation via a double-stranded endoribonuclease.

### RNA processing factors for the *rrn *operon may be limited in AS5^ox ^lines

When AS5 was expressed from within the *rrn *operon, several probes showed varied accumulation of precursor transcripts or processing intermediates compared to the WT. Some of these disparities may have arisen because RNA processing capacity had been exceeded, a phenomenon that has previously been observed. Transgenes inserted into the chloroplast *rrn *operon have been shown to accumulate >100-fold more RNA than an analogous nuclear (e.g. *aadA*) transformant due to plastid genome polyploidy [32,33]. Excess RNA produced by a chloroplast transgene would unlikely stimulate any increase in the nuclear-encoded and chloroplast-targeted proteins necessary for its maturation. For example, when the *clpP *5' UTR was overexpressed in tobacco chloroplasts a reduction in endogenous mature *clpP *mRNA was observed due to the limited availability of a *clpP*-specific mRNA maturation factor [34].

In this study, we observed a several-fold increase in transcription rates distal to the *aadA *insertion, which is substantial given that the *rrn *promoter is already the strongest in the chloroplast [35]. The most strongly affected gene in this region was *trnA*, most likely due to its proximity to the *aadA *promoter. Extra precursor accumulation from the *rrn *operon, however, probably does not contribute to the slow-growth phenotype of AS5^ox ^lines. Previous studies where transgenes were targeted to the *rrn *operon also resulted in substantial accumulation of polycistronic transcripts, but little effect on plant development and chloroplast protein expression was noted, other than the intended effects of the transgenes, such as increasing tolerance to abiotic stress [32,36,37]. However, in one case plant development was delayed [38].

Mature tRNA^Ile ^accumulation is decreased somewhat in the AS5^ox ^lines, while tRNA^Ala ^was not affected. We considered whether tRNA^Ile ^deficiency could explain the polysome shift (Figure 7) and slow growth phenotype. We concluded this is unlikely because the chloroplast encodes a second tRNA^Ile ^isoacceptor (CAU) within the inverted repeat region that could compensate for the decrease in the affected tRNA^Ile^-GAU using wobble base pairing, which is common in chloroplasts [39]. The accumulation of tRNA precursors can probably be ascribed to transgene sequences that interfere with RNA structures recognized by processing enzymes.

Multiple AS5-containing precursor transcripts also accumulated in the transgenic lines, while a family of smaller AS5 transcripts (< 400 nt) were detected by RNA gel blot for both WT and AS5^ox ^plants. The precursor transcripts are polycistronic intermediates, while the smaller transcripts may be a result of AS5 maturation or degradation. The AS5-containing polycistronic transcripts appear to be poor substrates for chloroplast RNA processing enzymes, although the smaller species exhibited a 3- to 10-fold greater accumulation in the AS5^ox ^lines than in the WT (Figure 4). This is far less than the qRT-PCR results, which showed a >500-fold overaccumulation of AS5 in the transgenic lines due to quantification of the very abundant polycistronic precursors in addition to the smaller AS5 species. These longer RNAs could have less biological activity than the small molecules in terms of 5S rRNA binding and/or destabilization, although a small proportion of them was found in the heavier fractions of polysome gradients (Figure 7).

### 5S rRNA stability is decreased in transgenic plants

Accumulation of mature rRNA transcripts differed in the AS5^ox ^lines. It was expected and observed that transcription of *rrn23 *and *rrn5 *would increase due to the position of the transgene promoter, and that *rrn16 *would be unaffected. However, there was only a minor (~1.5-fold) increase in mature 23S rRNA, no difference in 4.5S and 5S rRNAs, and a small decrease in 16S transcripts. The mechanisms surrounding rRNA degradation and ribosome copy control in plant organelles are poorly understood, although in bacteria it has been established that rRNAs accumulate in a molar ratio with their ribosomal protein counterparts, and if one or the other is in excess they are targeted for degradation [reviewed in [40]]. Bacterial rRNA turnover also occurs as a way to regulate translation during slow growth and under certain stress conditions, and to prevent the accumulation of misassembled ribosomes. The variability in mature rRNA accumulation in the AS5^ox ^lines is likely due to post-transcriptional mechanisms that equalize rRNA stoichiometry by degrading excess transcripts according to the bacterial paradigm. To this end, we speculate that AS5 may be regulating 5S accumulation, leading to degradation of excess 23S and 4.5S rRNAs. Crosstalk between chloroplast rRNAs was previously observed in the tomato *dcl *mutant, whose primary deficiency is in 4.5S rRNA, but also exhbited decreased polysomal loading of 5S rRNA [41].

### Native AS5 may be processed *in vivo*

Multiple AS5 species were detected in both WT and AS5^ox ^lines. Among ncRNAs, the existence of multiple transcripts is relatively common for eukaryotic miRNAs and their precursors [42]. The transcript profiles of organellar ncRNAs, however, have been characterized in only a few cases. In the case of chloroplast ncRNAs, both very small [22-55 nt; 10] and longer [400-650 nt; 43] transcripts have been verified by gel blots. In the latter case, three transcripts accumulated from the *ndhB *antisense strand. AS5 could be produced by transcriptional read-through from *trnN*, and/or from a promoter between *trnN *and AS5. Either would create longer precursors that could give rise to the observed species.

Further evidence that AS5 is processed comes from the EST database, which shows variable 5' and 3' ends from tomato AS5 transcripts (Figure 3A), something that is mirrored when AS5 ESTs are extracted for *Arabidopsis *(data not shown). However, ESTs are often derived from protocols that exclude smaller transcripts (< 200 nt), so AS5 transcripts such as the 70 nt species detected by RNA gel blot (Figure 3B) would not be found even if it were polyadenylated.

The 70 nt AS5 species is derived from the 5S-*trnR *intergenic region near the mature 5S rRNA 3' end, a region previously shown to be a site for endonuclease cleavage during 5S rRNA maturation [27]. This raises the possibility that some AS5 bands could be derived from cleavage of a longer AS5 species bound to its putative 5S rRNA target. Both 5S rRNA and the 5S-*trnR *intergenic region are predicted to form multiple stem-loops [44,45], which would interfere with full-length sense-antisense duplex formation and leave single-stranded regions available for ribonucleolytic cleavage, or promote cleavage by an RNase III-like activity. Partial duplexing between exposed loops or bulges is common for prokaryotic ncRNAs and their targets [46,47]. One example is binding of DsrA to its mRNA target, *rpoS*. This creates an RNase III processing site, which, following cleavage, produces stable transcripts of each [48].

Some AS5 transcripts may be degradation intermediates unrelated to 5S rRNA interactions. In prokaryotes, both bound and unbound ncRNAs are regulated by multiple ribonucleases, including RNase E, PNPase, RNase II and RNase III. For example, the bacterial ncRNA RNAI degradation intermediate is visible by RNA blot after RNase E cleavage near the 5' end. Following this, the transcript is polyadenylated and degraded by RNase II and PNPase [49]. Similarly, multiple transcripts are observed for the *Salmonella typhimurium *ncRNAs CsrA, CsrB, MicA, and SraL, which were determined to be degradation intermediates [50].

### AS5 may regulate 5S rRNA

As discussed above, our results lead to the hypothesis that at least a portion of overexpessed AS5 is binding to pre- and/or mature 5S rRNA to inhibit its maturation or incorporation into ribosomes, ultimately targeting it for degradation via an RNase E or RNase III-dependent pathway. RNase E is present in chloroplasts [51,52], and one RNase III-like protein has been detected to date [53]. Additional members of the prokaryotic RNase III family have also been predicted [54].

Based on bacterial data, 5S rRNA is thought to stabilize the 50S subunit, enhance peptidyl transferase activity, and assist in tRNA binding [45]. In *E. coli*, sequential deletion of 5S rRNA genes resulted in a slower growth rate [55], which parallels the slower growth of the AS5^ox ^lines where 5S rRNA availability may be limited by AS5. Mature 5S rRNA binds to ribosomal proteins L5 and L18, and this complex is incorporated into the pre-50S subunit, a process that may be facilitated by the RNA-binding proteins CSP41a and CSP41b [56,57]. Excess AS5 may be a competitive inhibitor of L5 and L18, preventing ribosomal incorporation.

Our working hypothesis is that AS5 is part of a quality/quantity control mechanism for 5S rRNA. While 5S rRNA has not been proposed as a regulatory target in chloroplasts, other examples do exist. In the case of nuclear-encoded 5S rRNA in *Xenopus *oocytes, the Ro protein associates specifically with 5S sequences that have extra 3' nucleotides and/or point mutations. These faulty 5S sequences are eventually degraded, and it is thought that binding of Ro acts as a chaperone for target recognition by ribonucleases [58]. A second regulatory mechanism occurs in *Arabidopsis *via a heterochromatic siRNA, siR1003. In this case, aberrant 5S transcripts extending into an intergenic spacer region are cleaved into siRNAs. These siRNAs regulate the methylation status of the 5S rDNA to prevent further accumulation of long 5S transcripts [59]. While these particular modes of 5S regulation could not occur in chloroplasts, they do suggest that organisms have evolved ways of detecting excess and/or faulty 5S rRNA.

## Conclusion

AS5 is one of the first natural chloroplast-encoded asRNAs to be investigated for a possible *cis *regulatory role in gene expression. This function is commonly observed for both prokaryotic and eukaryotic ncRNAs. Overexpression of AS5 led to slower growth, which we interpret as a pleiotropic effect of its interaction with 5S rRNA. AS5 may interact dynamically with 5S rRNA as influenced by the accumulation of other ribosomal transcripts and proteins. We hypothesize that the role of AS5 is to prevent the accumulation of misprocessed 5S rRNA, as well as to control its stoichiometry. Further studies are needed to determine the enzymes involved in the maturation and turnover of AS5, and other chloroplast-encoded asRNAs.

## Methods

### Nucleic Acid Manipulation

The transformation vector diagrammed in Figure 1A was obtained by modification of the plasmid ptrnI-RT [60]. Briefly, the T7G10 5' UTR was removed by digestion with *Asc*I and *Nde*I. The 450 bp AS5 sequence was PCR-amplified from total tobacco DNA with the primers AS5^ox ^5' and 3' (Additional File 1), incorporating *Nhe*I and *Cla*I restriction sites, respectively, then inserted into pCR2.1. The resulting plasmid was *Nhe*I-*Not*I digested, and ligated to the modified ptrnI-RT vector to yield the final pAS5OX plasmid.

### Chloroplast Transformation

Chloroplast transformants were obtained by bombardment of two week-old *Nicotiana tabacum cv. petite Havana *seedlings using 0.6 μm gold particles coated with pAS5OX [22,61]. Transformants were selected on RMOP-spectinomycin (500 mg L^-1^), then subjected to 3-4 rounds of regeneration on RMOP-spectinomycin/streptomycin (500 mg L^-1 ^ea.). AS5^ox ^transformants were monitored by PCR and DNA gel blot. Two homoplasmic lines were obtained (AS5^ox^-1 and -2), and transferred to MS-spectinomycin (500 mg L^-1^) for rooting. The transformants were grown on soil at 25°C with a 16-h light/dark photoperiod and the seeds collected. All subsequent analyses utilized homoplasmic AS5^ox ^plants germinated on soil.

### DNA Gel Blots

Leaf or callus tissue was ground in liquid nitrogen with a mortar and pestle. DNA was extracted using the DNeasy Plant Mini Kit (Qiagen, Valencia, CA) according to the manufacturer's instructions. Total DNA was digested with *Xho*I and *Hind*III and hybridized using the method of Church and Gilbert [62]. Primers *trnI-trnA *F and R (Additional File 1) were used to amplify a 250 bp region between the *trnI *3' exon and *trnA *intron, which was used as a template for probe synthesis as described below. The hybridized membrane was visualized using a Storm Scanner (Molecular Dynamics, Sunnyvale, CA).

### Phenotypic Analysis

Plants were grown in flats for two weeks on soil supplemented with osmocote fertilizer under a 16-h light/dark cycle, then transferred to two gallon pots with the same soil until flowering, when total chlorophyll and shoot height were determined (Table 1). For chlorophyll analysis, duplicate 0.1 cm^2 ^leaf discs were frozen in liquid nitrogen and ground in 1 mL of methanol using a Wheaton homogenizer. Cell debris was pelleted by centrifugation at 13,000 rpm for 3 min at 4°C, after which total chlorophyll was determined as previously described [63]. In a second analysis, plants were germinated and grown under the same conditions, but phenotypic measurements were taken 40 days after germination. Shoot height was measured from the top of the soil to the apical meristem. Internodes and leaves were counted starting at the base of the plant to the apical meristem. Internode circumference was measured midway between surrounding leaves. Leaf length/width were measured at the longest/widest points, and leaf weight was determined on a per area basis.

### RNA Isolation and qRT-PCR

Mature leaf tissue was ground in liquid nitrogen, and total RNA extracted using TRI reagent (Molecular Research Center, Cincinnati, OH) with minor modifications to the manufacturer's instructions. RNA was precipitated overnight with isopropanol at -20°C, and the pellet was washed with 75% ethanol and dissolved in water. For strand-specific cDNA synthesis, 1 μg of DNase-treated RNA was reverse transcribed with SuperScript III (Invitrogen, Carlsbad, CA) using the 3' qPCR gene-specific primers (Additional File 1). The qPCR reaction contained 1× Fast SYBR Green Master Mix (Applied Biosystems, Carlsbad, CA), 2 ng cDNA, and 300 nM of each primer, except for 18S rRNA (200 nM each primer), in a 20 μL volume. Amplification was done in a Bio-Rad CFX96 real-time PCR detection system (Hercules, CA) using the following two-step cycling conditions: initial denaturation at 95°C for 3 min, followed by 40 cycles at 95°C for 10S and 59°C for 30 s (+plate read), and a final incubation at 95°C for 10 s, after which a melt curve analysis was completed (59-95°C in 0.5°C steps) to ensure amplification specificity. Quantification and primer efficiencies were determined by comparison to four-step standard curves (0.04-10.00 ng cDNA in five-fold increments). Relative quantification compared to WT samples (given a reference value of 1) was achieved after normalization to 18S rRNA and GAPDH mRNA by the Bio-Rad CFX Manager software, taking into account differences in primer efficiencies. The final data is an average of three biological and at least two technical replicates.

### RNA Gel Blots

One microgram of total RNA per sample was separated in 1.2% agarose/formaldehyde gels, which were probed with either double-stranded DNA or single-stranded RNA probes as indicated in the Figure Legends. DNA probes were synthesized from 100 ng of PCR template, and hybridized to the RNA gel blot at 65°C according to Church and Gilbert [62]. RNA probes were made from 100 ng of PCR template containing a T7 promoter using T7 polymerase and 40 μCi α-^32^P-UTP, and then gel purified. For RNA probes, membranes were pre-hybridized for 6 hrs in 50% formamide, 20× SSC, 2% bovine serum albumin, 0.6% SDS and 200 μg mL^-1 ^denatured salmon sperm DNA at 65°C, after which the RNA probe was denatured and allowed to hybridize overnight at 65°C. Membranes were then washed at 65°C twice for 5 min in 1× SSC and 0.6% SDS, followed by two 20 min washes in 0.3× SSC and 0.6% SDS. For polysome analysis, 200 mg of leaf tissue was extracted and fractionated through a 15-55% sucrose density step gradient [64], and the RNA extracted and analyzed as described above. To analyze small RNA fragments, 5 μg of total RNA was separated in a 10% polyacrylamide gel, electroblotted onto Hybond-N+ nylon membrane (GE Healthcare, Piscataway, NJ) in 1× TBE buffer using a TE 77 semi-dry transfer apparatus (GE Healthcare), UV-crosslinked, and probed as described above.

### Chloroplast Isolation and Run-on Transcription

Chloroplasts from WT and AS5^ox ^lines were isolated from 40 g of leaf tissue as previously described [65], with minor modifications. Intact chloroplasts were collected from the interface of a 40-80% sucrose density gradient. The final chloroplast pellet was washed with two volumes of IC buffer (50 mM HEPES, pH 8.0 and 0.33 M sorbitol) and resuspended in 1 mL IC buffer. Plastid number was determined with a hemacytometer. Transcription was initiated by the addition of 2.5 × 10^6 ^plastids per 25 μL reaction containing 4 μCi μL^-1 ^α-^32^P-UTP that was equilibrated at 25°C [66]. The reaction was terminated after 5 min by the addition of an equal volume of stop solution (5% SDS, 50 mM Tris-HCl pH 8.0, and 25 mM EDTA), and then extracted with phenol:chloroform. The supernatant was treated with DNase, followed by a second phenol:chloroform extraction and ethanol precipitation. The pellet was resuspended in 50 μL of TE, and unincorporated nucleotides were removed on a Sephadex G-25 column. The final RNA sample was denatured at 65°C for 15 min, and then used as a probe for DNA slot blots [67]. PCR products representing *psbA*, *rrn16*, *rrn23*, *rrn5-trnR*, *aadA*, and *cat *(negative control) were denatured and spotted onto Hybond-N+ membrane by vacuum filtration using a Hybri-Slot Manifold (Bethesda Research Laboratories, Carlsbad, CA). The membrane was UV-crosslinked, and then pre-hybridized for 12 hrs at 65°C in hybridization solution (6× SSC, 5× Denhardt's solution, 0.5% SDS, and 40 μg mL^-1 ^denatured salmon sperm DNA). The probe was allowed to hybridize for 24 hrs, after which the membrane was washed twice for 30 min in wash buffer 1 (2× SSC, 0.1% SDS) and once for 30 min in wash buffer 2 (0.5× SSC, 0.1% SDS) at 65°C [68]. The membrane was visualized and quantified using a Storm scanner (GE Healthcare).

## Authors' contributions

AMH carried out the experimental procedures, and ZEH assisted in the phenotypic analyses in Figure 2 and Table 1. DBS participated in planning the experiments and revising the manuscript. All authors read and approved the final manuscript.

## Supplementary Material

Additional file 1 **PCR Primers**. Click here for file
