# Supplementary material for: Overexpression of a natural chloroplast-encoded antisense RNA in tobacco destabilizes 5S rRNA and retards plant growth
Source: BMC Plant Biol. 2010 Sep 29;10:213. doi: 10.1186/1471-2229-10-213 (PMC3017836; doi:10.1186/1471-2229-10-213)
Supplement: Additional file 1 — PCR Primers. [file 1471-2229-10-213-S1.PDF]

Additional File 1 – Primer Table

Primer	Sequence (5'→3')
AS5^{ox} 5'	**GCTAGC**TCCCGATCATGATTTTCCTACC
AS5^{ox} 3'	**ATCGAT**CTCTACTGCGGTGACGATACTG
trnI-trnA F	ACGGGCGAGGTCTCTGGTTCA
trnI-trnA R	GCACGTTTCGGTCCTCTTCC
5S qPCR 5'	TCCTAGGCGTAGAGGAAC
5S qPCR 3'	CTGGCGTCGAGCTATTTTTC
trnR qPCR 5'	GCTCAGAGGATTAGAGCACG
trnR qPCR 3'	GTGGGCGAGGAGGGATTCG
AS5 qPCR 5'	CCGTCTCCACTGGATCTGTT
AS5 qPCR 3'	CCCAATTGTGACATCCCTTC
18S qPCR 5'	CAGCTCGTGTCGTGAGATGT
18S qPCR 3'	TTCTCCTTAGGCGCATGTCT
GAPDH qPCR 5'	GGTGGTGCCAAGAAAGTCAT
GAPDH qPCR 3'	TGAGCTCGGGCTTGTATTCT
AS5 3' T7	***TAATACGACTCACTATAGGG***GCTTAACACCTCTCATTC
P1	GTGGGCGAGGAGGGATTCG
P2	CCGTCTCCACTGGATCTGTT
P3	GAAGGGATGTCACAATTGGG
16S 5'	GTGAGTAACGCGTAAGAACC
16S 3'	AATTAAACCACATGCTCCAC
trnI 5'	GGCTATTAGCTCAGTGG
trnI 3'	TGGGCCATCCTGGACTT
aadA 5'	CGACTCAACTATCAGAGG
aadA 3'	CGTAGTGGACAAATTCTTCC
trnA 5'	GGATATAGCTCAGTTGGTAGA
trnA 3'	TGGAGATAAGCGGACTCGAACCG
23S 5'	CATCTAGGGGTAAAGCACTG
23S 3'	GATGCTTTCAGCAGTTATCC
4.5S 5'	CGAGACGAGCCGTTTATCAT
4.5S 3'	TCTACCGGTCTGTTAGGATGC
rbcL 5'	CCGCCTCATGGGATCCAA
rbcL 3'	CTTATCCAAAACGTCCAC
psbA 5'	CCTATGGGGTCGCTTCTGTA
psbA 3'	AGCTGCAACAGGAGCTGAAT
cat 5'	CCAGCGGCATCAGCACCTTGTCG
cat 3'	TCTTGCCCGCCTGATGAATGCTC
aadA R	CGGAGCCGTACAAATGTA

Note: AS5^{ox} 5’ and 3’ boldface indicate *Nhe*I and *Cla*I restriction sites, respectively; the AS5 3’ T7 boldface italics is the T7 recognition sequence
